# A propensity score-matched analysis between patients with high hip dislocation after childhood pyogenic infection and Crowe IV developmental dysplasia of the hip in total hip arthroplasty with subtrochanteric shortening osteotomy

**DOI:** 10.1186/s13018-020-01947-5

**Published:** 2020-09-17

**Authors:** Enze Zhao, Zunhan Liu, Zichuan Ding, Zhenyu Luo, Hao Li, Zongke Zhou

**Affiliations:** Department of Orthopedics, West China Hospital/West China School of Medicine, Sichuan University, 37# Wuhou Guoxue Road, Chengdu, People’s Republic of China

**Keywords:** Total hip arthroplasty, Subtrochanteric shortening osteotomy, High hip dislocation, Childhood pyogenic infection, Developmental dysplasia of the hip

## Abstract

**Background:**

Whether satisfactory clinical and radiological outcomes of total hip arthroplasty (THA) with subtrochanteric shortening osteotomy (SSO) in high hip dislocation after childhood pyogenic infection can be achieved as in Crowe IV developmental dysplasia of the hip (DDH) remains unclear.

**Methods:**

Between September 2009 and December 2016, 151 primary THAs performed at our institution using similar SSO technique and prosthetic design were retrospectively reviewed. After excluding patients who met exclusion criteria, 29 patients were identified as high dislocation (Crowe IV) after childhood infection (HDACI) and 107 as Crowe IV developmental dysplasia of the hip (DDH). Propensity score matching was used to select 29 Crowe IV DDH patients as a control group for the HDACI group with comparable preoperative conditions. Clinical and radiological outcomes and complication were compared and analyzed. The mean follow-up duration of the 2 groups was 5.0 years.

**Results:**

The mean Harris hip score (HHS) and the mean score in range of motion (ROM) domain of the modified Merle d’Aubigné-Postel (MAP) were 84.6 and 4.5 in the HDACI group, compared with 88.3 and 4.9 in the DDH group; there was significant difference between the 2 groups in these parameters (*P* = 0.015 and 0.035, respectively). Meanwhile, in the HDACI group, the median time of osteotomy union was 4 months and osteotomy nonunion rate was 3%; no significant difference was detected in the median time of osteotomy union and osteotomy nonunion rate between the 2 groups (*P* = 0.388 and 1.000, respectively). And no significant difference was found in the rate of complications between two groups.

**Conclusions:**

HDACI patients who received THA combined with SSO could achieve similar satisfactory results as DDH patients in Crowe type IV. The fixation technique of autogenous cortical bone struts had a positive influence on osteotomy healing of SSO in this specific setting.

## Introduction

Pyogenic arthritis of the hip is still a common disease for children in less-developed countries [1]. High hip dislocation is a catastrophic sequela of hip pyogenic arthritis [2, 3]. Chronic high hip dislocation could affect patients’ normal activities and cause severe pain of hip and low back. Total hip arthroplasty (THA) remains the gold standard for pain relief and hip functional recovery in patients with high dislocation (Crowe IV) after childhood infection (HDACI).

Restoration of the hip rotation center into the true acetabulum in THA has been reported to ensure a more durable prosthesis [4–9]. However, it remains an intractable problem in patients with chronic high hip dislocation. Subtrochanteric shortening osteotomy (SSO) is a useful technique to facilitate the restoration of the anatomic hip rotation center without stretching the nerves, despite pathoanatomy of severely dysplastic hips and extensive contracture in the periarticular soft tissues. Subtrochanteric shortening osteotomy has been reported marked outcomes in THA of Crowe IV DDH [6, 10–12]. However, to our knowledge, limited studies in the literature have reported the results of THA with SSO in patients with high hip dislocation after childhood pyogenic infection [13–18]. Moreover, these prior reports have no control group and include cases without receiving SSO or osteotomy site fixation [13–18].

The objective of this article was to study the result of THA with SSO in the setting of high hip dislocation secondary to childhood pyogenic infection by comparing the clinical and radiological outcomes with Crowe IV DDH patients who underwent THA with SSO. Specifically, we asked (1) whether HDACI patients have similar functional recovery after THA with SSO as Crowe IV DDH patients, (2) whether osteotomy with autogenous cortical bone struts fixation had a satisfied union rate and union time of osteotomy in this specific setting compared with Crowe IV DDH patients, and (3) whether HDACI patients have an increased complication risk compared with Crowe IV DDH patients after THA with SSO.

## Materials and methods

After approval from our institutional review board of West China Hospital, we retrospectively review 151 consecutive patients (160 hips) who underwent primary THA with SSO between 2009 and 2016. Of these, 5 patients (5 hips) died for reasons unrelated to the surgery, and 5 patients (5 hips) were lost and could not be contacted by telephone or e-mail. Exclusion criteria removed patients that underwent THA with SSO due to hip pyogenic arthritis secondary to previous hip surgeries (2 hips) or tuberculosis hip infection (3 hips). The remaining cases were identified as 29 HDACI cases and 107 Crowe IV DDH cases with a minimum 3-year follow-up. Hips with evidence of previous pyogenic arthritis, including history (i.e., clear evidence of previous infection of the hip joint from medical or surgical data), the clinical or radiographic features of previous infection (i.e., the old presence of sinus tract), and prior bacteriologic findings, were deemed as the HDACI group. The infecting organism of the original infection (i.e., open incision and drainage in 20 hips and aspirate in 9 hips) was *Staphylococcus aureus* in 27 hips (93.2%), *Streptococcus* in 1 (3.4%), and *Escherichia coli* in 1 (3.4%). Of 29 cases with a history of childhood hip pyogenic infection, all of them were treated with irrigation, debridement, and intravenous antibiotic therapy, and only one patient underwent extra open reduction. The mean age of infection diagnosis was 7.8 ± 6.3 years. We support the finding of the earlier reports that the previous infected hips that had remained quiescent for<10 years may have increased risk of reactivation of the prior infection after THA and all previous infective hips met the criterion of remained quiescent for>10 years with the mean 35-year quiescent period [19, 20]. According to age, gender, BMI, ASA score, preoperative leg length discrepancy (LLD), and follow-up duration, propensity score matching was used to select 29 DDH patients as a control group (DDH group) for the HDACI group with comparable preoperative conditions. The two groups were compared to study the clinical and radiological outcomes of THA with SSO.

### Surgical technique

All surgeries were designed with transparencies and used cementless femoral and acetabular prosthesis. To restore hip rotation center into the true acetabulum and to improve leg length discrepancy, the amount of femoral shortening was measured and calculated. All legs were lengthened by no more than 3–4 cm to avoid nerve stretching.

All patients received general anesthesia, a posterolateral approach and similar technique of transverse subtrochanteric shortening osteotomy as Yasgur et al. described [21]. After total capsulectomy were performed, intraoperative swab and tissue cultures were conducted due to previous hip pyogenic arthritis and then removal of the femoral head, fibrous scar tissue, and osteophyte were conducted to uncover the true acetabulum. Soft tissue release was performed in all hips. Normally, a transverse femoral osteotomy was conducted approximately 1–2 cm distal to the lesser trochanter to remove the obstruction of the proximal part of the femur. If proximal femoral canal deformity was presented, SSO was conducted at the proximal deformity. The short section of the vastus lateralis was lifted or split to approach the subtrochanteric region. After transversal osteotomy, the proximal femoral fragment was translated anteriorly to access to the true acetabulum. Then the acetabulum was reamed gradually to prepare the medial wall of the true acetabulum. Press-fit technique was used in all implantation of the acetabular. If there were severe bone defects of acetabulum or insufficient coverage, a structural autograft or titanium alloy (Ti-alloy) mesh combined with bulk bone grafting from the excised femoral head was used.

**Fig. 1:**
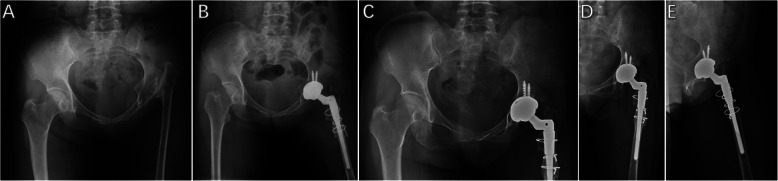
Radiographs of a 50-year-old female patient with high hip dislocation secondary to childhood pyogenic infection that had a quiescent period of 46 years between infection and left THA. **a** Preoperative AP pelvic radiograph. **b** Immediately postoperative AP pelvic radiograph after left THA with subtrochanteric shortening osteotomy. **c** Postoperative AP pelvic radiograph 6 years after THA. **d** Postoperative AP and **e** oblique hip radiographs 6 years after THA

**Fig. 2:**
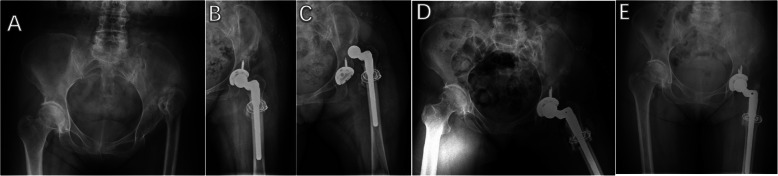
Radiographs of a 48-year-old female patient with high hip dislocation secondary to childhood pyogenic infection that had a quiescent period of 40 years between infection and left THA. **a** Preoperative AP pelvic radiograph. **b** Immediately postoperative AP hip radiograph. **c** Dislocation of the left hip 6 days postoperatively. **d** Postoperative dislocation treated with closed reduction. **e** Postoperative AP pelvic radiographs 4 years after THA

Then the second transverse subtrochanteric osteotomy was performed to shorten the femur as preoperative planned. The femoral axial intramedullary reaming process was performed sequentially until the appropriate femoral component size was achieved. If a trial hip reduction could not be accomplished after the first cut of osteotomy, the additional transverse osteotomy was gradually conducted to achieve appropriate hip reduction in the true acetabulum. A mean length of subtrochanteric segmental resection was 2.8 cm (range, 1.8–4.6 cm) in the HDACI group and 3.0 cm (range, 1.9–5.5 cm) in the DDH group. The straight stem (S-ROM, DePuy) was inserted into the femoral intramedullary cavity through the osteotomy site in all patients while ensuring that 15° to 20° of anteversion of the femoral component was placed by adjusting rotational alignment of the 2 fragments. In all patients, the longitudinally autogenous cortical bone struts from the resected cylindrical femoral fragment were used to fix the osteotomy site with cables to enhance biologic recovery and autogenous morselized cancellous bone from the resected femoral head was taken to fill the space at the osteotomy site (Figs. 1 and 2). Comparisons of component information and surgical characteristics are summarized in Table 2.

All patients were encouraged to carry out isometric exercises and active limb mobilization on bed immediately after surgery. All patients were allowed partial weight-bearing with crutches as tolerated on the second post-operative day and progressed to full weight-bearing after 6 weeks depending on the stability of the femoral stem and osseous healing at osteotomy site [22].

### Clinical evaluations

Clinical evaluations were conducted preoperatively and 3 weeks, 8 weeks, 16 weeks, and 6 months postoperatively and annually thereafter until the last follow-up. Patients with unusual symptoms or abnormal radiographic findings were conducted with more frequent evaluations. Clinical scores were derived using the Harris hip score (HHS) system, the modified Merle d’Aubigné-Postel (MAP) hip score (including pain, motion, and gait function), and the 12-item short-form health survey (SF-12) [23–25]. In addition to the recognized patient-reported outcomes, all pre- and post-operative evaluations involving Trendelenburg test status were also recorded. The leg length discrepancy (LLD) was measured from the anterior superior iliac spine to the medial malleolus representing the length discrepancy of lower extremities and was recorded pre- and post-operatively. Limb lengthening was defined as the difference in the preoperative and postoperative LLD. The individual blood volume was calculated by the Nadler et al. method, and the individual total blood loss was calculated using the difference in hemoglobin levels introduced by the Good et al. [26, 27]. Complications including recurrent infection, periprosthetic fracture, implant instability, nerve palsy, dislocation, nonunion of osteotomy, heterotopic ossification, and revision were also recorded.

### Radiographic measurements

Radiographic analyses were carried out based on serial standardized anteroposterior and lateral radiographs of the affected hip to assess osteotomy healing, osteotomy migration, femoral component stability, component loosening, and heterotopic ossification by two authors who had not participated in the surgery. Periprosthetic radiolucency around acetabulum and femoral components was recorded as described by Delee and Charnley and Gruen et al., respectively [28, 29]. Femoral component loosening was diagnosed by radiographic presence described by Engh et al. [30]. The acetabular component loosening was diagnosed by radiographic presence of progressive radiolucent lines of > 2 mm around the inserted cup, or migration, or a change in the position of the cup based on the 3 zones around the acetabular cup [31]. Time to osteotomy union was recorded depending on radiographic presence described by Masonis et al. [32]. The heterotopic ossification was assessed by using the method described by Brooker et al. [33]. Subsidence of the femoral component was assessed as the alteration in the distance from the center of the superomedial tip of the femoral stem to the most proximal point on the lesser trochanter and the change of > 5 mm was considered meaningful. Osteointegration of the femoral prosthesis was classified (i.e., bone ingrown, stable fibrous ingrown, or unstable) as described by Engh et al. [34].

### Statistical analysis

Propensity scores depending on logistic regression was used to match the HDACI group and DDH group. Student’s *t* test and the Wilcoxon rank-sum test were used to analyzed continuous variables while comparing between two groups. The paired *t* test was used to analyze the preoperative and postoperative continuous variables for each group. Categorical values were analyzed using the chi-squared or Fisher’s exact test. All statistical analyses were performed using SPSS v25.0 (IBM, Armonk, NY). *P* values < 0.05 were considered statistically significant.

## Results

The comparisons of the demographic data between two groups are summarized in Table 1; there were no significant differences between the 2 groups before the surgery. The mean preoperative LLD was 4.8 cm in the HDACI group and 4.1 cm in the DDH group (*P* = 0.638). All patients had preoperative whole blood cell counts, C-reactive protein limits, erythrocyte sedimentation rates, and IL-6 levels that were within the normal ranges. All intraoperative examinations were found to be negative for infection including intraoperatively frozen sections, synovial fluid, synovial tissues, and excised specimen cultures. No significant difference was found in perioperative blood loss and operation time between the 2 groups (Table 2).

**Table 1:** Baseline characteristics of all recruited patients

Variable	HDACI (*n* = 29)	DDH (*n* = 29)	*P* value
Age (years)	42.8 ± 10.8	41.7 ± 12.5	0.720
Female gender	18 (62%)	18 (62%)	1.000
Body mass index (kg/m^2^)	22.9 ± 2.7	22.3 ± 3.2	0.403
ASA score			
1	26 (90%)	26 (90%)	1.000
2	3 (10%)	3 (10%)	1.000
Preoperative LLD (cm)	4.8 ± 1.7	4.1 ± 1.6	0.638
Average follow-up (years)	5.0 ± 1.8	5.0 ± 1.8	1.000
Trendelenburg sign (no. of hips)	29	29	1.000
Preoperative Harris hip score	42.9 ± 9.9	44.1 ± 9.4	0.654
Modified MAP			
Mean in points (mean ± SD)	6.5 ± 2.0	6.6 ± 2.1	0.949
Pain	2.1 ± 0.9	2.2 ± 0.8	0.649
Walking	1.9 ± 0.5	1.8 ± 0.5	0.612
ROM	2.5 ± 0.9	2.5 ± 0.9	1.000
SF-12			
PCS	10.5 ± 1.5	10.8 ± 1.6	0.451
MCS	13.7 ± 1.9	14.1 ± 1.4	0.347

*HDACI* high dislocation after childhood infection, *DDH* developmental dysplasia of the hip, *ASA* American Society of Anesthesiologists, *LLD* leg length discrepancy, *MAP* Merle d’Aubigné-Postel, *ROM* range of motion, *SF-12* 12-item short-form health survey questionnaire, *MCS* mental component summary, *PCS* physical component summary, *SD* standard deviation

**Table 2:** Component information and surgical characteristics

Variable	HDACI (*n* = 29)	DDH (*n* = 29)	*P* value
Cup diameter (mm)			0.793
38	1	0	
40	3	2	
42	1	2	
44	12	12	
46	3	7	
48	6	4	
50	0	0	
52	3	2	
Head diameter (mm)			0.758
22	5	5	
28	15	18	
32	6	5	
36	3	1	
Types of bearing surface (no. of hips)			0.913
Ceramic-on-ceramic bearing	18	18	
Ceramic-on-polyethylene bearing	5	6	
Metal-on-polyethylene bearing	6	5	
Length of femoral resection (cm)	2.8 ± 0.9	3.0 ± 1.2	0.396
Operation time (min)	157.6 ± 24.2	146.1 ± 27.3	0.095
Perioperative blood loss (mL)	457 ± 170	382 ± 166	0.094

*HDACI* high dislocation after childhood infection, *DDH* developmental dysplasia of the hip

The postoperative clinical outcomes are summarized in Table 3. The mean Harris hip score, modified MAP score, SF-12 score, and Trendelenburg sign significantly improved postoperatively compared with preoperatively in each group. At the last follow-up, the mean postoperative HHS was 84.6 in the HDACI group and 88.3 in the DDH group (*P* = 0.015). Although no significant difference was seen between the 2 groups in the mean modified MAP score (*P* = 0.149), there was significant difference between the 2 groups in the mean score in range of motion domain of the modified MAP with 4.5 in the HDACI group and 4.9 in the DDH group (*P* = 0.035). The mean postoperative LLD was 1.2 cm in the HDACI group and 1.0 cm in the DDH group (*P* = 0.459).

**Table 3:** Postoperative clinical outcomes

Variable	HDACI (*n* = 29)	DDH (*n* = 29)	*P* value
Postoperative LLD (cm)	1.2 ± 0.9	1.0 ± 0.7	0.459
Trendelenburg sign (no. of hips)	2	0	0.491
Postoperative Harris hip score	84.6 ± 6.2	88.3 ± 5.2	**0.015**
Postoperative modified MAP			
Mean in points (mean ± SD)	14.7 ± 1.6	15.3 ± 1.6	0.149
Pain	5.7 ± 0.5	5.7 ± 0.6	0.821
Walking	4.6 ± 0.6	4.8 ± 0.6	0.147
ROM	4.5 ± 0.6	4.9 ± 0.6	**0.035**
SF-12			
PCS	20.3 ± 1.6	20.9 ± 1.5	0.125
MCS	23.5 ± 1.7	24.1 ± 1.8	0.171

*P* < 0.05 indicated significant differences

*HDACI* high dislocation after childhood infection, *DDH* developmental dysplasia of the hip, *LLD* leg length discrepancy, *MAP* Merle d’Aubigné-Postel, *ROM* range of motion, *SF-12* 12-item short-form health survey questionnaire, *MCS* mental component summary, *PCS* physical component summary, *SD* standard deviation

The radiographic results are summarized in Table 4. There was no significant difference in the median time for union of the osteotomy site between two groups (median 4 vs 4 months, *P* = 0.388). At the latest follow-up, in the HDACI group, a nonprogressive radiolucent line (< 2 mm) was seen in 2 hips around the acetabular cup (1 in zone I and 1 in zone II, respectively) and in 2 hips around the femoral stem (1 in zone 1 and 1 in zone 7). And a progressive radiolucent line was seen in one hip around undersized femoral stem of the HDACI group with nonunion of the osteotomy site, aseptic loosening, stem subsidence, and femoral stem breakage. Stem revision was performed with a larger monoblock cementless femoral stem 1 year after the index arthroplasty. In the DDH group, there were 2 hips presenting nonprogressive radiolucent line around the acetabular cup (2 in zone II) and 2 hips presenting nonprogressive radiolucent line around the femoral stem (1 in zone 1 and 1 in zone 7). There was no significant difference between the 2 groups in stem stability according to the Engh classification (*P* = 0.670) [34]. In comparison of the two groups, heterotopic ossification was not significantly different (6 in the HDACI group and 4 in DDH group, *P* = 0.487) on the last follow-up radiographs. According to the Brooker classification system asymptomatic class-I heterotopic ossification was observed in 4 hips of the HDACI group and 3 hips of the DDH group and class-II was observed in 2 hips of the HDACI group and one hip of the DDH group.

**Table 4:** Radiographic Outcomes

Parameters	HDACI (*n* = 29)	DDH (*n* = 29)	*P* value
Time for union of SSO (mo)^a^	4 (3-10) ^b^	4 (2-10)	0.388
Radiolucency around cup	2 (7%)	2 (7%)	1.000
Radiolucency around stem	3 (10%)	2 (7%)	1.000
Stem subsidence	1 (3%)	0 (0%)	1.000
Stem stability			0.670
Bone ingrowth	25(87%)	27(93%)	
Fibrous stable	3 (10%)	2 (7%)	
Loosening	1 (3%)	0 (0%)	
Heterotopic ossification	6 (21%)	4 (14%)	0.487

*HDACI* high dislocation after childhood infection, *DDH* developmental dysplasia of the hip, *SSO* subtrochanteric shortening osteotomy

^a^The values are given as the median with the range in parentheses

^b^One outliner is removed from the data

No periprosthetic infection was observed during the follow-up in each group (Table 5). Two hips underwent surgical site infection in the HDACI group and recovered after debridement, intravenous, and oral antibiotic therapy. There was one femoral nerve palsy combined with sciatic nerve palsy and 2 femoral nerve palsies in the HDACI group, and one sciatic nerve palsy in the DDH group, all of which recovered fully after neurotrophic therapy and rehabilitation training within 6 months without further sequelae. Seven hips in the HDACI group and six hips in DDH group occurred intraoperative femoral fracture, all of which were fixed with cerclage cable. Two patients in the HDACI group and one patient in the DDH group experienced postoperative dislocation, which was treated with closed reduction (Fig. 2). In each group, there was one patient underwent postoperative dislocation with treatment of open reduction without additional recurrence. As aforementioned, there was one patient in the HDACI group with nonunion of the osteotomy site, aseptic loosening, and femoral stem crack. Femoral revisions were performed in the patient and showed excellent outcome at last follow-up.

**Table 5:** Complications

Complications	HDACI (*n* = 29)	DDH (*n* = 29)	*P* value
Surgical site infection	2 (7%)	0 (0%)	0.491
Periprosthetic infection	0 (0%)	0 (0%)	1.000
Sciatic nerve palsy	1 (3%)	1 (3%)	1.000
Femoral nerve palsy	3 (10%)	0 (0%)	0.237
Intraoperative periprosthetic femoral fracture	7 (24%)	6 (21%)	0.753
Dislocation	3 (10%)	2 (7%)	1.000
Aseptic loosening	1 (3%)	0 (0%)	1.000
Osteotomy site nonunion	1 (3%)	0 (0%)	1.000

*HDACI* high dislocation after childhood infection, *DDH* developmental dysplasia of the hip

## Discussion

The restoration of the hip rotation center into the true acetabulum promotes long-term durability with lower loosening and aseptic revision rates in patients who received THA [35]. In THA for severe DDH, SSO has been shown to facilitate restoration of the anatomical hip rotation center and provide excellent exposure of the true acetabulum [36]. However, for patients with high hip dislocation after childhood pyogenic infection, limited studies have reported series of who received THA and SSO using a single method to fix the osteotomy site [13, 15–17]. Furthermore, to our knowledge, only one study has compared the results of THA and SSO with multiple fixation between these 2 etiologies [14]. Therefore, we attempt to conduct the present study by comparing the clinical and radiographic outcomes of THA and transverse SSO with autogenous cortical bone struts fixation between HDACI and Crowe IV DDH patients.

The challenge of THA in chronic dislocation depends on the severity of anatomic deformation including high hip center, small femoral canal, rotational deformity of the proximal femur, thickening of the capsule, dysfunction of the transverse abductor, thickening of the iliopsoas, contracture of the hamstring, adductor and rectus femoris muscles, and contracture of the sciatic nerve and deep femoral artery, which also influence the postoperative results [3, 37]. We found that the mean postoperative Harris hip score and modified MAP score in our HDACI group are comparable with prior studies [13–17]. However, lower mean score in postoperative Harris hip score and range of motion domain of the modified MAP was identified in the HDACI group compared with the DDH group in the present study. Similar results have been reported in several literatures [14, 15]. The possible interpretations may include the following: first, besides aforementioned anatomic deformation of chronic dislocation, previous infection may result in more severe soft tissue contractures, changed positions of the femoral nerve and vessels, and fibrotic muscles; second, a number of scar tissue repair after previous surgical procedures led to limited range of motion; and third, to avoid damaging deformed neurovascular structures and enhance recovery after surgery, we choose limited soft tissue release rather than widely exposure as far as possible during the index surgery.

In this study, we used similar technique in all groups, including fixation of osteotomy site with cables and the longitudinally split removed autogenous segment. An important finding of this study is that SSO with autogenous cortical bone struts fixation has a predictable and high rate of osteotomy healing within a relatively rapid time in the setting of HDACI. The nonunion rate of SSO in both groups is comparable with previous studies of DDH patients reporting a nonunion rate that ranged from 0 to 11% [21, 38, 39]. There was only 1 case of osteotomy nonunion in the HDACI group, which healed successfully after stem reimplantation surgery with satisfying outcomes during the follow-up. In the remaining 28 HDACI patients, osteotomy healing occurred at a median time of 4 months, comparable with that in DDH group. Park et al. [14] compared the results of THA with SSO between 25 HDACI patients and 25 Crowe IV DDH patients and reported longer median time for union of SSO and higher reoperation rate of additional fixation in the HDACI patients. However, 40% and 32% HDACI patients were performed with non-fixed SSO and additional longitudinal osteotomy, respectively, which may confound their results. Using plate and screw for fixation of transverse osteotomy were also reported and showed satisfying outcomes [6, 21]. Nevertheless, the periosteal damage may have a delaying effect on osteotomy healing while applying a plate and screw. Catma et al. reported that fixation of the longitudinally split removed autogenous segment with cables may provide competent rotational stability and resulted in early union at the osteotomy site [40]. Therefore, although the S-ROM prosthesis can serve as an intramedullary nail splinting the osteotomy site and SSO without any fixation was reported with satisfying outcomes in DDH patients [5, 41], we recommend routinely using autogenous cortical bone struts on the site of SSO with cerclage wiring in HDACI patients who underwent THA and SSO.

Concerns of increased the complications rate and recurrent infection always exist when reconstructing hips with previous hip septic arthritis. Kim et al. reported that the previous infected hips that had remained quiescent for < 10 years may have increased risk of reactivation of the prior infection after THA [19, 20]. In the present study, all previous infective hips met the criterion of over 10-year quiescent period without positive finding for evidence of active infection and no recurrent infection happened during the follow-up. However, there are two cases in the HDACI group who experienced surgical site infection with different infecting organism from the original infection after the index surgery. One of the cases, a 44-year-old male, had hip joint infection (*Staphylococcus aureus*) remained quiescent for 40 years at the time of THA and SSO. Three years after the index surgery, a subcutaneous abscess (*Nontuberculous mycobacteria*) appeared in the surgical site. Fortunately, the abscess did not communicate with the articular cavity and the infection did not recur after treatment with focal debridement, intravenous, and oral antibiotic therapy. Therefore, bacteriological samples should be collected extensively to maximize the opportunities of discovering residual bacteria in order to prevent catastrophic consequences such as periprosthetic joint infection among these susceptible patients with a history of childhood pyogenic infection.

Higher rates of postoperative complications in the HDACI group were mostly caused by nerve palsy and dislocation rather than reactivation of the prior infection. It was reported that the risk of neuralgic traction injury increases when restoration of the anatomic hip rotation center requires limb lengthening in excess of 3–4 cm [42, 43]. In the present study, 3 patients (10%) in the HDACI group experienced temporary femoral nerve paralysis postoperatively without further sequelae and the mean leg lengthening of the three was 2.7 cm. Although both groups had overall low incidence of nerve palsy, there was a trend toward a higher rate in the HDACI group. Due to contracture and adhesions in the HDACI group, the nerve extensibility is worse than that in Crowe IV DDH patients so that the safe range of limb lengthening for non-pyogenic DDH patients may also cause nerve injury in HDACI patients. Therefore, care should be taken to conduct meticulous surgical manipulation including proper retractor placement, technique of effective soft tissue release, and relatively less limb lengthening in this group of patients.

Several limitations exist in the present study. First, the retrospective nature of the present study decreased the level of evidence. Second, sample size was relatively small. Third, mean 5-year follow-up is relatively short-term, and longer-term follow-up and larger sample size would provide more meaningful data. Fourth, the operations were conducted by 5 senior joint surgeons; therefore, the differences in surgical manipulations between surgeons may cause potential statistical bias. Despite these limitations, this study involved the relatively large of such sample sizes to date comparing the clinical and radiological outcomes of THA with SSO between the HDACI and Crowe IV DDH, two uncommon diagnoses. In addition, different surgeons made the study results easier to replicate and generalize. The importance of this study is that it demonstrated the efficacy and safety of SSO with predictable osteotomy healing in the setting of high hip dislocation after childhood pyogenic infection.

## Conclusions

Although HDACI patients who received THA combined with SSO had relatively lower clinical scores compared with Crowe IV DDH patients, satisfactory results could also be achieved in HDACI patients. Furthermore, our study also suggests that the fixation technique of autogenous cortical bone struts may enhance the union of osteotomy site in HDACI patients who received THA and SSO. Therefore, THA and SSO combined with the fixation technique of autogenous cortical bone struts remains a good choice in HDACI patients, not at the cost of increasing the rate of overall complications and the risk of recurrent infection.

## Data Availability

The datasets analyzed during the current study are available from the corresponding author on reasonable request.

## Abbreviations

DDH: Developmental dysplasia of the hip
HDACI: High dislocation after childhood infection
ASA: American Society of Anesthesiologists
LLD: Leg length discrepancy
MAP: Merle d’Aubigné-Postel
ROM: Range of motion
SF-12: 12-item short-form health survey questionnaire
MCS: Mental component summary
PCS: Physical component summary
SD: Standard deviation
